# Supplementary material for: Matching sensor ontologies through siamese neural networks without using reference alignment
Source: PeerJ Comput Sci. 2021 Jun 18;7:e602. doi: 10.7717/peerj-cs.602 (PMC8237319; doi:10.7717/peerj-cs.602)
Supplement: Supplemental Information 1 [file peerj-cs-07-602-s001.zip › 253/refalign.html]

# (level 0) Alignment

## Source: http://oaei.ontologymatching.org/2011/benchmarks/101/onto.rdf

## Target: http://oaei.ontologymatching.org/2011/benchmarks/253/onto.rdf

## Correspondences

type = sqbdzg
:   1.0

howPublished = sdqz
:   1.0

periodicity = sdxsndxsqg
:   1.0

proceedings = zassdzadb
:   1.0

volume = zsbdgz
:   1.0

annote = dsqnhza
:   1.0

PersonList = dsqdbz
:   1.0

month = asndzsd
:   1.0

copyright = zdzdzd
:   1.0

Unpublished = zeadza
:   1.0

address = qzd
:   1.0

Address = qzddj
:   1.0

chapter = dsazdjz
:   1.0

Chapter = dzqndbzq
:   1.0

editor = dzajj
:   1.0

InBook = vccfsq
:   1.0

Date = dzshyuua
:   1.0

series = dszbnz
:   1.0

PageRange = dnqsb
:   1.0

date = sqndsqgy
:   1.0

title = dznbaln
:   1.0

Booklet = zdzaudbd
:   1.0

numberOrVolume = dzezd
:   1.0

LectureNotes = scds
:   1.0

url = zdsznbadzagh
:   1.0

MastersThesis = xsqlknk
:   1.0

organizer = sdnsbh
:   1.0

mrNumber = dszbgz
:   1.0

pages = dzayue
:   1.0

TechReport = sdcsqhyz
:   1.0

reviewed = dsqndbsqx
:   1.0

startPage = sqnhdbdj
:   1.0

edition = sdsqddz
:   1.0

lccn = dzandzah
:   1.0

affiliation = zadzqbsdg
:   1.0

institution = hsgiuyza
:   1.0

year = zdsnzsdh
:   1.0

isPartOf = EzGNplyLBiS
:   1.0

organization = sqdndvb
:   1.0

country = zadszabnds
:   1.0

publisher = sddnsnqdb
:   1.0

school = dzbn
:   1.0

Misc = wxsdxqsdh
:   1.0

collection = sqnbyz
:   1.0

Collection = sqxsqkd
:   1.0

isbn = szdnzak
:   1.0

abstract = dsqndsz
:   1.0

directors = zdnzqnd
:   1.0

Academic = zdazsx
:   1.0

location = zdjznzn
:   1.0

MotionPicture = dscdscg
:   1.0

Article = hazdn
:   1.0

Informal = qsdsnbsqd
:   1.0

price = dsq
:   1.0

name = dszabdza
:   1.0

event = zadzjadns
:   1.0

state = zdnzadh
:   1.0

Book = zajdhqs
:   1.0

book = sznsbq
:   1.0

day = dznadzh
:   1.0

School = zadazxn
:   1.0

shortName = dsza
:   1.0

PhdThesis = zqdzqzai
:   1.0

Proceedings = zdqssqdb
:   1.0

number = ndsbzh
:   1.0

issue = sqnzkzn
:   1.0

Reference = sqdsq
:   1.0

endPage = mkalnshsq
:   1.0

InCollection = dcsqdcsqd
:   1.0

firstPublished = sqdnbs
:   1.0

author = zand
:   1.0

Report = dqzdxdcsqj
:   1.0

note = zdsnsqdv
:   1.0

humanCreator = sbqgzga
:   1.0

Conference = zqedzbx
:   1.0

Part = sqdsopq
:   1.0

Publisher = zauio
:   1.0

contract = zandsbh
:   1.0

Manual = dcsqdsq
:   1.0

key = zdzbh
:   1.0

InProceedings = deqdxcsqcsq
:   1.0

Journal = qsdsquj
:   1.0

journal = sxqsnbvsq
:   1.0

Monograph = dzajndsq
:   1.0

keywords = zqdszh
:   1.0

issn = zadsznad
:   1.0

contents = zdnzdzh
:   1.0

city = zdzndh
:   1.0

Institution = izxnquo
:   1.0

Deliverable = azzauhuie
:   1.0

size = dzsndzah
:   1.0

chapters = iNi4b8XV
:   1.0

parts = TfniY
:   1.0

communications = PrSGUs
:   1.0

articles = YuEma
:   1.0

lastName = lastName
:   1.0

language = sqkxndc
:   1.0
